# Genome-wide identification and expression analysis of *CPP*-like gene family in *Triticum aestivum* L. under different hormone and stress conditions

**DOI:** 10.1515/biol-2022-0051

**Published:** 2022-05-18

**Authors:** Uzair Ullah, Zeeshan Ali Buttar, Abdullah Shalmani, Izhar Muhammad, Aziz Ud-Din, Hamid Ali

**Affiliations:** Department of Biotechnology and Genetic Engineering, University Mansehra, Dhodial, Pakistan; The Collaborative Innovation Center for Grain Crops, Henan Agricultural University, Zhengzhou, China; College of Life Sciences, Northwest A & F University, Xianyang, China

**Keywords:** wheat (*Triticum aestivum* L.), rice (*Oryza sativa*), CPP, IAA, ACC

## Abstract

The *CPP*-like plant‐specific transcription factor has a prominent role in plant development and growth through cell division and differential activities. However, little information is available about the *CPP* gene family in *Triticum aestivum* L. Herein, we identified 37 and 11 *CPP* genes in the wheat and rice genome databases, respectively. The phylogeny of the CPP protein-like family members was further divided into five subfamilies based on structural similarities and phenotypic functional diversities. The *in silico* expression analysis showed that *CPP* genes are highly expressed in some tissues, such as shoot apex, shoot, leaf, leaf sheath, and microspore. Furthermore, the qRT-PCR found higher expression for *TaCPP* gene family members in leaf, leaf blade, young spike, mature spike, and differential expression patterns under abiotic stresses, including heat, drought, salt, and hormonal treatment, such as indole acetic acid and 1-aminocyclopropane-1 carboxylic acid. We found that *CPP* gene family members are mostly located in the nucleus after infiltrating the *CPP5-1B-GFP* and *TaCPP11-3B-GFP* into tobacco leaves. The overexpression of the *TaCPP5-1D* gene revealed that the *CPP* gene positively regulates the germanium, shoot, and root activities in *Arabidopsis*. The *TaCPP5-1D*-overexpressed plants showed less anti-oxidative sensitivity under drought stress conditions. These results demonstrated that TaCPP5-1D protein has a crucial contribution by interacting with TaCPP11-3B protein in maintaining stress homeostasis under the natural and unfavorable environmental conditions for growth, development, and stress resistance activities. Therefore, this study could be used as pioneer knowledge to further investigate the function of *CPP* genes in plant growth and development.

## 1 Introduction

Previous studies reported that transcription factors (TFs) play a vital role in regulating gene expression at the mRNA transcript level. The *CPP*-like gene family is also known as cysteine-rich polycomb-like proteins, classified as important regulatory proteins mainly found in all plants and animals apart from yeast [1]. CPP TFs are derived from plants that consist of 1 or 2 cysteine-rich conserved motifs recognized as CXC domains (CXCX4CX3YCXCX6CX3CXCX2C) [1,2]. The CPP proteins are classified into two different categories based on their location composed of different amino acids, which controlled diverse functions in plants [3]. Furthermore, the CXC domain and the sequences that linked the CXC domains are highly conserved across all living organisms [4,5]. Furthermore, it has been reported that highly conserved inter-domain and domain sequences may help the *CPP* gene family to control gene expression by sticky DNA along with CXC motifs [6,7].

Recent studies confirmed the CXC domains’ functional role within CPP TFs to promote the expression of the target genes linked with plant growth, development, and stress responses [6,7]. For instance, the CXC domain-containing CBBP protein (also known as b1-repeat binding protein) may bind to a certain region within the b1 tandem repeat sequence to regulate para-mutation in maize [8]. Furthermore, the CPP-like TF played a vital role in cell division and flower development by negatively regulating the MYB3R1 in *Arabidopsis* [7,9,10]. CPP TFs also perform a diverse function, particularly to regulate cell division and the development of several tissues [9,10]. In addition, the first TF, namely TSO1, was identified in *Arabidopsis* by using map-based cloning, which showed high expression level in the flower ovule and microspore development, suggesting the contribution of the CPP genes in the development of reproductive organs [7,9,10,11,12]. It was also noted that STO1 could promote cellular expansion by altering the communication of the neighboring cell development, cytokinesis, and karyokinesis [7,11,12]. The CPP transcription-like protein, named CPP1, regulates the Gmlbc3 protein in root development in soybean [13].

Recent studies have reported that several TFs promote plant resistance against several adverse environmental conditions. For instance, it has been found that BBX and YABBY TFs perform a vital role in plant stress biology [14,15,16]. The biotic stresses greatly affect the function of CPP TFs in plant growth and developmental processes. For example, under heat stress, the transcript level of the CPP genes was promoted in the root [17]. The transcript level of the ZmCPP TF was increased under heat, cold, and drought stress in maize [18]. A recent study found that the CPP-like TFs displayed diverse expression patterns in different tissues in the tea plant, suggesting the involvement of CPP TFs in a wide range of physiological and developmental processes [2]. Therefore, we assumed that CPP regulatory proteins might potentially contribute to the plant developmental and growth processes in beneficial coordination with several abiotic stresses.

Undoubtedly, in agriculture, wheat and rice are regarded as utmost important cereal crops. Therefore, it is important to identify and study the TFs that could enhance the resistance against various unfavorable conditions. So far, the CPP-like TFs have been reported in maize, *Arabidopsis*, soybean, rice, cucumber, and tea plants [1,2,17,18]. However, no comprehensive study has investigated the CPP-like TF family members and their evolutionary relationship and expression profile under different hormonal and abiotic stresses in wheat. The present study evaluated the role of biological evolution in the expansion of the CPP-like TFs in rice, wheat, and *Arabidopsis* and the potential role of CPP proteins in the plant growth and development under various adverse environmental factors overexpressing the *TaCPP5-1D* gene in *Arabidopsis*. The results will provide a solid foundation for exploring the molecular mechanism of the CPP-like TFs in plants.

## 2 Materials and methods

### 2.1 Identification and physicochemical properties of CPP genes

To find CPP TFs in rice and wheat, we first retrieved the CPP TFs from the *Arabidopsis* genome (https://www.arabidopsis.org/). The identified *Arabidopsis* CPP proteins were then blasted in the rice genome database (http://rice.plantbiology.msu.edu/) and the wheat genome database (www.wheatgenome.org) using BlastP. To avoid the possible loss of the CPP gene due to the CPP incomplete domain, a local BLASTP was performed with a cutoff of 1 × 10^−5^. After that, all protein sequences were examined to confirm the presence of the CPP-conserved domain through SMART (http://smart.embl-heidelberg.de/) [19], Inter Pro Scan program (https://www.ebi.ac.uk/interpro/), Conservative Domain Database (CDD) (http://www.ncbi.nlm.nih.gov/cdd/), and Scan Prosite (https://prosite.expasy.org/scanprosite/). In the present study, the physicochemical characteristics, including isoelectric point (pI), molecular weight (MW), GRAVY, and the up–down strand of the identified CPP TFs, were carried out using ExPASy online server (https://www.expasy.org) [20].

### 2.2 Chromosomal location and multiple sequence alignment of *CPP* genes

Rice (http://rice.plantbiology.msu.edu/) and wheat genome databases (www.wheatgenome.org) were used to find information about the chromosome location of identified CPP TFs in wheat and rice. A diagram of the physical location of the identified CPP genes was constructed using MapDraw in excel 2007. The naming of the identified *CPP* genes was performed based on their location on the chromosome. Multiple sequence alignments were performed to identify the conserved regions in the CPP proteins using DNAMAN software (Lynnon Corporation, Canada, https://www.lynnon.com). The Logos of the conserved region were constructed through an online Web logo server (https://weblogo.berkeley.edu/logo.cgi) [21].

### 2.3 Phylogenetic relationships, gene structures, motif, and duplication analysis

To investigate the evolutionary relationship of the CPP proteins among the plant, the multiple sequence alignments of the rice, wheat, and *Arabidopsis*-identified CPP proteins were performed using the ClustalW v2.0 online package (http://www.ebi.ac.uk). Then, the aligned sequence of the identified CPP proteins of rice, wheat, and *Arabidopsis* was used for the phylogenetic analysis using iTOL (https://itol.embl.de/) [22] and MEGA software [23] via maximum likelihood methods with 1,000 bootstrap values. The genomic and coding sequence of the identified *CPP* genes was collected from their respective genome databases to evaluate the intron/exon distribution of the *CPP* genes through Gene Structure Display online Server (http://gsds1.cbi.pku.edu.cn/) [24]. The conserved motifs of CPP protein sequences were analyzed using the online Multiple Expectation Maximization for Motif Elucidation platform (version 4.12.0) (https://meme-suite.org/meme/doc/meme.html) [25], with default parameter. The maximum number of motifs was 10, and the optimum motif width was set to >6 and <200. Several studies have found that tandem and segmental gene duplication contributes significantly to the growth of genes in a living organism throughout evolution [15,16,26]. Therefore, the present study also explored the relationship between duplication and CPP TFs’ expansion in rice, and wheat using the Plant Genome Duplication Database (http://chibba.agtec.uga.edu/duplication/). The circus software (http://circos.ca/) [27] was used to draw the lines among the syntenic blocks that were obtained from the Plant Genome Duplication Database (http://chibba.agtec.uga.edu/duplication/).

### 2.4 Developmental expression profile of CPP genes

The GENEVESTIGATOR V3 database (https://genevestigator.com/gv/) [28] was used to assess the transcript level of the *CPP* gene family at different developmental stages and tissues. The gene IDs of the identified *CPP* genes were pasted in the search area of these available microarray data search engines, and the data were collected in the form of a heat map. The different colors were used to distinguish the upregulated and downregulated genes in the heat map. The wheat plants were grown in the field of Hazara University for the transcript evaluation of CPP TFs in the diverse organs using semi-quantitative real-time polymerase chain reaction (qRT-PCR). The tissues, including leaf, root, shoot, stem, node, internode, leaf sheath, and leaf flag, were harvested at several stages, such as the booting stage, seedling stage, and heading stage. The tissues were stored at −80 for further analysis.

### 2.5 Expression profile analysis of *CPP* genes under different abiotic and hormonal stresses

The biological materials, such as seeds, were obtained from the Department of Genetics, Hazara University. The uniform and healthy seeds were grown in the field or the growth chamber in pods under optimum environmental conditions. The optimum condition for wheat growth was set as follows: daytime 16 h and night 8 h, the temperature should be between 21 and 25°C, light intensity should be 800 µmol m^−2 ^s^−1^, and the relative humidity should be 60–70%. The hormonal application and abiotic stresses were performed according to our previously published paper in environmental research [29]. Briefly, for the heat stress treatment, the seedlings at the four-leaf stage were subjected to 40°C temperature with 60% humidity and a 16 h photoperiod in the growth chamber under fluorescent light for 24 h. For the dehydration of 20% polyethylene glycol (PEG 6000), the solution was purified through an ion-exchange column to remove any impurities and filtered using Miracloth (22–25 µm; Thomas Scientific, Swedesboro, NJ, USA). Salt (200 mM NaCl) was prepared from stock solution by dissolving in water. Then, seedlings were submerged in 20% PEG 6000 and 200 mM NaCl solution for drought and salt treatments, respectively. The final hormonal concentration of 1-aminocyclopropane-1 carboxylic acid (ACC) deaminase (50 µM) and indole acetic acid (IAA) (50 µM) was prepared from stock solutions; after the addition of wetting agent Tween-20 at 0.05% (v/v), the individual hormone was sprayed on 2-week-old wheat plants. The samples were collected at different time points for expression analysis.

### 2.6 Quantitative PCR analysis

The total RNA was extracted from all the samples by using the cetyltrimethylammonium bromide method [30]. The samples were run on 2% agarose gels to examine the intensity of ribosomal RNA bands, degraded products, and gDNA contamination. The residual genomic DNA was removed by treating the RNA samples with RNase-free DNase. The cDNA was synthesized through the PrimeScript RT Reagent Kit with gDNA Eraser (Takara Bio, Shiga, Japan) following the manufacturer’s instructions. All the primers were designed from wheat *CPP* sequences for real-time PCR using primer 6.0 (Table S2). Each primer pair was examined through standard real-time polymerase chain reaction (RT-PCR) to confirm the size of the amplified product through 1% agarose gel electrophoresis. Real-time PCR was carried out in an iCycler iQ Real-Time PCR Detection System (Bio-Rad). Each reaction consisted of 5 μL of SYBR Premix ExTaq (Takara, Kyoto, Japan), 2 μL of cDNA samples, and 0.5 μL of each primer (10 μM) and 2 μL of ddH_2_O in a reaction system of 10 μL. The thermal cycle was as follows: 95°C for 3 min, followed by 40 cycles at 94°C for 15 s, 62°C for 20 s, and 72°C for 20 s. The melting curve analysis was performed directly after real-time PCR to verify the presence of gene-specific PCR products. This analysis was done at 94°C for 15 s, followed by a constant increase from 60 to 95°C at a 2% ramp rate. The wheat actin gene was used as an internal control and served as a standard gene for normalizing all mRNA expression levels. The relative amount of template present in each PCR amplification mixture was evaluated using the 2^−ΔΔCt^ method.

### 2.7 Sub-cellular location and protein–protein interaction

The sub-cellular location was performed by cloning the CDS sequences of the *TaCPP5-1D* and *TaCPP11-3B* genes into the *pCAMBIA-1302* vector. The vectors were then transformed into *Agrobacterium tumefaciens* using the electric shock method. The vectors were then infiltrated into tobacco leaves, and the result was checked using the confocal microscope. The full-length CDS region of the *TaCPP5-1D* and *TaCPP11-3B* was ligated into different N-terminal and C-terminal of the luciferase reporter gene LUC, respectively. The constructed vectors were then co-infiltrated into tobacco leaves for firefly luciferase complementation imaging (LCI) assay. The co-expressed leaves were analyzed for the LUC activity at 60 h after co-infiltration through a plant living molecular marker imaging system (Lumazone Pylon 2048B, Princeton, USA). The primers used in the present study are listed in Table S2.

### 2.8 Construction of TaCPP5-1D *Arabidopsis* transgenic plants

The CDS region of the *TaCPP5-1D* gene was ligated into *pCAMBIA-1302*, and then the constructed vector was transformed into *A. tumefaciens*. The agrobacterium-mediated floral dipping method was used to transform the constructed vector into *Arabidopsis* wild-type (WT) Columbia. The ½ Murashige and Skoog (MS) medium containing 30 mg L^−1^ hygromycin was used to screen the T_0_ transgenic *Arabidopsis* plant. The seedlings with true green leaves were considered transformants. A total of three independent homozygous T3 lines (OE-1, OE-2, and OE-3) were used for further study. The transcript level of the three independent homozygous T3 lines (L1, L2, and L3) is presented in Figure 8a. All the primers used in this study are listed in Table S3.

### 2.9 Evaluation of germination, root, shoot, and antioxidant activities of overexpressed and WT plants

For germination, root, and shoot activities, the transgenic seeds were grown on the ½ MS medium containing 250 mM inositol. The germination rate was counted based on the appearance of an embryonic axis protrusion. For root and shoot activities, 2-week-old plants were used. The drought stress was performed by withholding the water for 2 weeks, and the samples were collected for antioxidant analysis. The superoxide dismutase (SOD), peroxidase (POD), catalase (CAT), and ascorbate peroxidase (APX) activities and malonaldehyde (MDA) content were evaluated by the previously described method [31].

### 2.10 Data analysis

The SPSS software was used to perform the statistical analysis. The mean and standard error were used for the graphs. The GraphPad Prism was used to construct the graphs.

## 3 Results

### 3.1 Identification of *CPP* genes in *Triticum aestivum* L. and *Oryza sativa*

In the present study, 37 CPP protein sequences were retrieved from the *T. aestivum* L. genome database. The nomenclature of all the *CPP* genes was performed based on their chromosome location and was named *TaCPP1* to *TaCPP17* (Table 1). We also found a total of 11 *CPP* genes in the rice genome, which were named *OsCPP1* to *OsCPP11* (Table 1). The family-specific domains, such as the CXC domain of the *CPP*-like gene family, were confirmed through the conserved domain, Pfam, and SMART databases. Based on the genome size, we found that *TaCPP15-5D* (9585 bp) was the largest, whereas *TaCPP14-4D* (963 bp) was the smallest *CCP* gene (Table 1). Based on the CDS and amino acid, the *TaCPP7-1D* and *TaCPP14-4D* were the largest and smallest CPP members, respectively (Table 1). We found that most of the identified *CPP* gene family members were located on the reversed strand in the rice and wheat genome. The physicochemical characteristics including MW, pI, amino acid composition, gravy, aliphatic index (AI), and instability index (II) of *CPP*-like gene family members were studied through the EXPASY PROTOPARAM http://www.expasy.org/tools/protparam.html online tool (Table S1). The range of MW was 14.28 (TaCPP11-3A) to 98.82 kDa (TaCPP3-1A) for the identified CPP-like proteins in rice and wheat. Most of the *CPP* gene family members were basic because their pI was higher than 7 (Table S1). The S (Ser) is the most abundant amino acid of the CPP proteins, followed by C (Cys), P (Pro), and A (Ala). The GRAVY value of all the identified rice and wheat CPP proteins is negative, suggesting that all the CPP proteins are non-polar and hydrophobic (Table S1). Moreover, the AI ranged from 30.31 (OsCPP8) to 75.95 (TaCPP9-2A) for the identified CPP proteins (Table S1). Based on the II, we found that the II value of all the CPP proteins was higher than 40, signifying that all the CPP proteins are unstable apart from TaCPP3-1B, TaCPP7-1D, and TaCPP8-1B.

**Table 1:** Identification and nomenclature of the identified CPP proteins in rice and wheat genome.

Name	Gene ID	Gene location	AA	CDS	Genomic	Strand
TaCPP1-1A	TraesCS1A02G327100.1	1A:516780762-516783619	372	1512	4058	R
TaCPP1-1B	TraesCS1B02G340500.1	1B:568536481-568539365	374	1522	4085	R
TaCPP1-1D	TraesCS1D02G329200.1	1D:420546981-420549825	374	1460	4045	R
TaCPP2-1B	TraesCS1B02G411800.1	1B:637913058-637915448	362	789	3591	R
TaCPP3-1A	TraesCS1A02G427700.1	1A:581879170-581880757	202	609	2788	F
TaCPP3-1B	TraesCS1B02G423800.1	1B:646682762-646687533	219	1293	5972	F
TaCPP4-1D	TraesCS1D02G393200.1	1D:462965510-462969112	180	1218	4803	R
TaCPP5-1B	TraesCS1B02G423900.1	1B:646775147-646776631	228	898	2685	F
TaCPP5-1D	TraesCS1D02G400400.1	1D:466604136-466605574	233	904	2639	F
TaCPP6-1A	TraesCS1A02G428000.2	1A:581957739-581959345	303	912	2807	F
TaCPP6-1B	TraesCS1B02G462600.1	1B:674816626-674821589	316	1530	6164	F
TaCPP7-1B	TraesCS1B02G462800.1	1B:674933401-674934839	235	918	2639	F
TaCPP7-1D	TraesCS1D02G437400.1	1D:484793121-484797317	230	1414	5397	F
TaCPP8-1B	TraesCS1B02G462900.1	1B:675227333-675228275	136	411	2144	R
TaCPP9-2A	TraesCS2A02G122300.2	2A:71665769-71671190	588	2324	6621	R
TaCPP9-2B	TraesCS2B02G144400.1	2B:110479459-110485095	589	2394	6640	R
TaCPP9-2D	TraesCS2D02G125000.1	2D:73099327-73104463	588	2213	6336	R
TaCPP10-2D	TraesCS2D02G563300.1	2D:634645178-634647593	264	795	3616	F
TaCPP11-3A	TraesCS3A02G219300.1	3A:402135204-402136682	242	729	2680	R
TaCPP11-3B	TraesCS3B02G249700.1	3B:397677642-397679106	261	786	2666	R
TaCPP11-3D	TraesCS3D02G233200.1	3D:321869369-321873110	243	2034	4442	F
TaCPP12-3A	TraesCS3A02G304800.1	3A:540959694-540968654	615	2518	963	F
TaCPP12-3B	TraesCS3B02G331700.1	3B:536986657-536994307	615	2485	8852	R
TaCPP12-3D	TraesCS3D02G297000.1	3D:410666651-410674769	615	2445	9320	R
TaCPP13-4A	TraesCS4A02G284600.1	4A:590993901-590999432	793	2841	6733	R
TaCPP13-4B	TraesCS4B02G028500.1	4B:21187118-21192530	789	2711	6611	F
TaCPP13-4D	TraesCS4D02G026100.1	4D:11364255-11369752	787	2799	6694	F
TaCPP14-4D	TraesCS4D02G184600.2	4D:322849931-322857603	890	3204	8873	F
TaCPP15-5A	TraesCS5A02G053800.1	5A:48916968-48924858	765	2812	9089	F
TaCPP15-5B	TraesCS5B02G062400.1	5B:70090784-70098944	765	2962	9360	R
TaCPP15-5D	TraesCS5D02G065300.1	5D:60631878-60640264	765	3047	9585	F
TaCPP16-5A	TraesCS5A02G054200.1	5A:50743216-50746791	357	1656	4774	F
TaCPP16-5B	TraesCS5B02G062200.1	5B:69855996-69859508	360	1298	4712	R
TaCPP16-5D	TraesCS5D02G065500.1	5D:60896691-60899936	359	1345	4444	F
TaCPP17-7A	TraesCS7A02G341100.1	7A:501056605-501062654	486	2071	7250	F
TaCPP17-7B	TraesCS7B02G242300.1	7B:450563457-450569975	485	2185	7719	F
TaCPP17-7D	TraesCS7D02G338500.1	7D:431930000-431936466	486	2147	7668	R
OsCPP1	LOC_Os01g55580.1	1: 32016622-32022401	619	1860	6980	R
OsCPP2	LOC_Os02g17460.1	2: 10042478-10049191	497	1494	7914	R
OsCPP3	LOC_Os03g43730.1	3: 24454953-24461662	757	2274	7910	R
OsCPP4	LOC_Os04g09560.1	4: 5130826-5132553	147	444	2928	R
OsCPP5	LOC_Os05g43380.1	5: 25224822-25227403	374	1125	3725	R
OsCPP6	LOC_Os05g51040.1	5: 29292436-29293646	194	585	1557	F
OsCPP7	LOC_Os06g22670.1	6: 13182291-13189126	518	1557	2411	R
OsCPP8	LOC_Os07g07974.1	7: 4,020265-4,027484	782	2349	8036	F
OsCPP9	LOC_Os08g28214.1	8: 17214129-17221249	598	1796	8420	R
OsCPP10	LOC_Os12g41210.1	12: 25553542-25557389	407	1224	5048	R
OsCPP11	LOC_Os12g41230.1	12: 25561750-25569052	670	2283	8503	R

AA, amino acid; CDS, coding sequence.

### 3.2 Evolutionary relationship, motifs analysis, and gene structure of *CPP* gene family members

**Figure 1:**
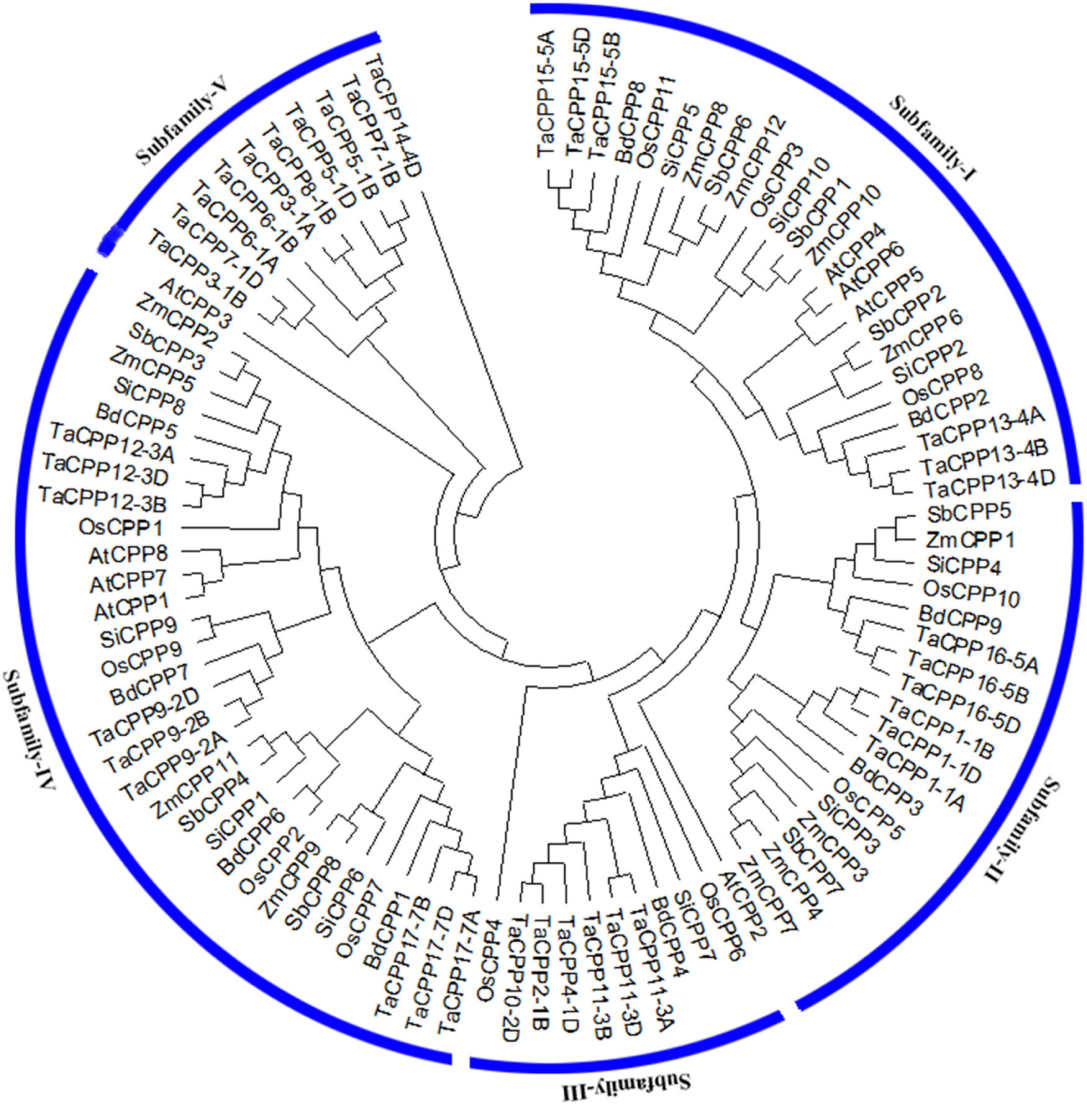
The phylogenetic tree of the CPP proteins between *Arabidopsis*, rice, wheat, maize, stiff brome, millet, and sorghum. The phylogenetic analysis divided the CPP gene family members into five subfamilies based on their physiochemical properties, intron–exon, and motif arrangement.

The identified *CPP* proteins from *Arabidopsis*, wheat, rice, maize, millet, sorghum, and stiff brome were used to construct a maximum likelihood phylogenetic tree (Figure 1), which provides more insight and knowledge about the evolutionary history CPP gene family in plants. The phylogenetic relationship divided the identified CPP proteins into five subfamilies (Figure 1). The highest number of CPP proteins was found in subfamily-IV, followed by subfamily-I and -II. The lowest number of CPP proteins was detected in subfamily-V and -III (Figure 1). We found that the distributions of CPP proteins into different subfamilies mainly occurred due to their physicochemical properties, intron–exon distribution, and motif arrangement. For instance, we found that the larger size *CPP* gene family members were clustered in subfamily-I, whereas the medium size CPP gene family members were found in subfamily-IV (Figure S1). Similarly, the smallest size *CPP* gene family members were noted in subfamily-II, -III, and -V. Based on the intron–exon distribution, we found that the *CPP* gene family members with a high number of exons (ten or more exons) were divided into subfamily-I. Most of the *CPP* genes possessing eight exons were found in subfamily-III. Similarly, six and five exons containing *CPP* genes were observed in subfamily-II and -IV, respectively (Figure S1). In subfamily-V, two to five exons were observed for most of the *CPP* gene family members apart from *TaCPP14-4D.* Furthermore, the *CPP* gene family members shared a quite similar motif distribution in each subfamily. For example, the CPP proteins possessing six to seven motifs were found in subfamily-I and -III, the CPP proteins having five motifs were clustered into subfamily-II, four motifs containing CPP proteins were detected in subfamily-IV, and two to three motifs possessing CPP proteins were divided into subfamily-V (Figure S1). Based on these results, we hypothesized that *CPP* gene family members shared conserved gene structure and common ancestor during biological evolution in plants. The intron–exon distribution was diverse among the identified CPP proteins (Figure S1). The highest numbers of exons (seventeen exons) were noted for *TaCPP14-4D* genes, whereas the lowest numbers of exons were observed for the *TaCPP6-1B* gene. Similarly, we found a total of ten motifs in the identified CPP proteins, which were named motif-1–10 (Figure S1). Motif-2 was the frequently repeated motif, followed by motif-7 and motif-3 in the identified CCP proteins in the studied plants. Moreover, motif-5 was the least repeated motif, followed by motif-6 and motif-8, respectively.

### 3.3 Chromosomal location and duplication of *CPP* gene family members

The *CPP* genes are unevenly distributed on the chromosomes in the genome of the wheat and rice based on the chromosome location and annotation information. The identified 11 *CPP* gene family members were located on 9 out of the 12 chromosomes in the rice genome. Among them, chromosomes 1, 2, 3, 4, 6, 7, and 8 each had one *OsCPP* gene, whereas chromosomes 5 and 12 shared two *CPP* genes in the rice genome (Figure S2a). In wheat, the identified *CPP* genes were distributed on all the chromosomes apart from chromosomes 6A, B, and D (Figure S2b). The highest number of *CPP* genes is seven *CPP* gene family members detected on chromosome-1B in the wheat genome. Only one *CPP* gene was distributed on each chromosome-2A, -4A, -7A, -2B, -4B, -7B, and -7D in the wheat genome. Similarly, we found two *CPP* genes on each chromosome-3A, -5A, -3B, -5B, -2D, -3D, -4D, and -5D (Figure S2b). The second-highest number of *CPP* gene (four) family members was observed on chromosome-1D, followed by chromosome-1A (shared three *CPP* gene family members) in the wheat genome.

**Figure 2:**
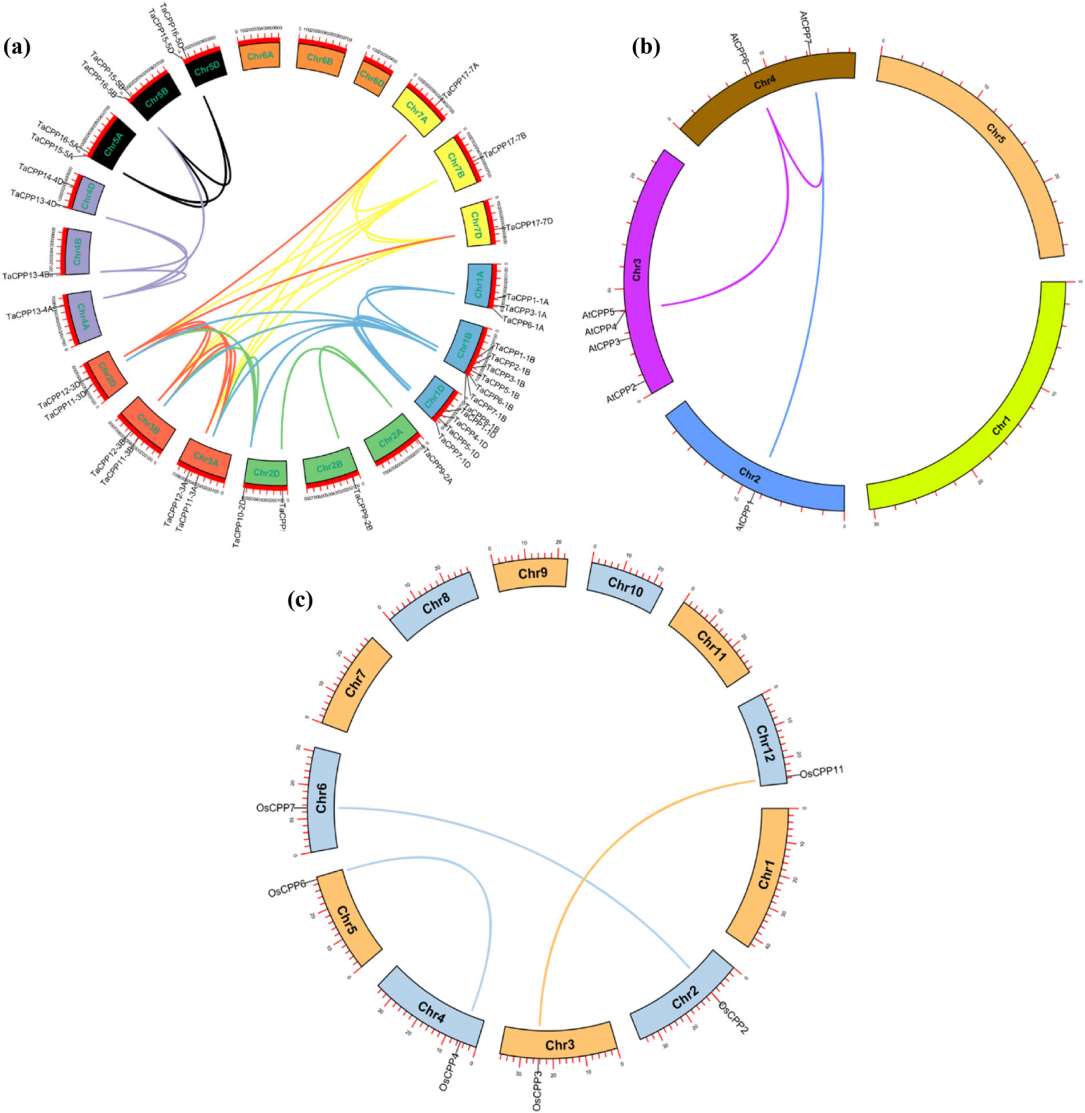
Genomic distribution of *TaCPP*, *OsCPP*, and *AtCPP* genes and gene homology analysis in rice, wheat, and *Arabidopsis*. The duplicated CPP gene pairs were found on the different chromosomes in rice, wheat, and *Arabidopsis*.

It has been reported that the plant genomes were widely composed of several segmental and duplicated genes. Therefore, we conducted a duplications analysis of the identified *CPP* gene family members in the studied plants to evaluate the role of duplication in expanding the *CPP* gene family in plants (Figure 2). We found several segmental duplicated pairs in the wheat genome for the *CPP* gene family members. A total of three segmental duplicated *CPP* genes were detected in the rice genome. Similarly, three segmental duplicated CPP pairs were observed in the *Arabidopsis* genome. All these results suggest that segmental duplication processes may play an important role in expanding the *CPP* gene family during biological evolution in plants.

### 3.4 Expression profiling of *CPP* genes in various tissues

**Figure 3:**
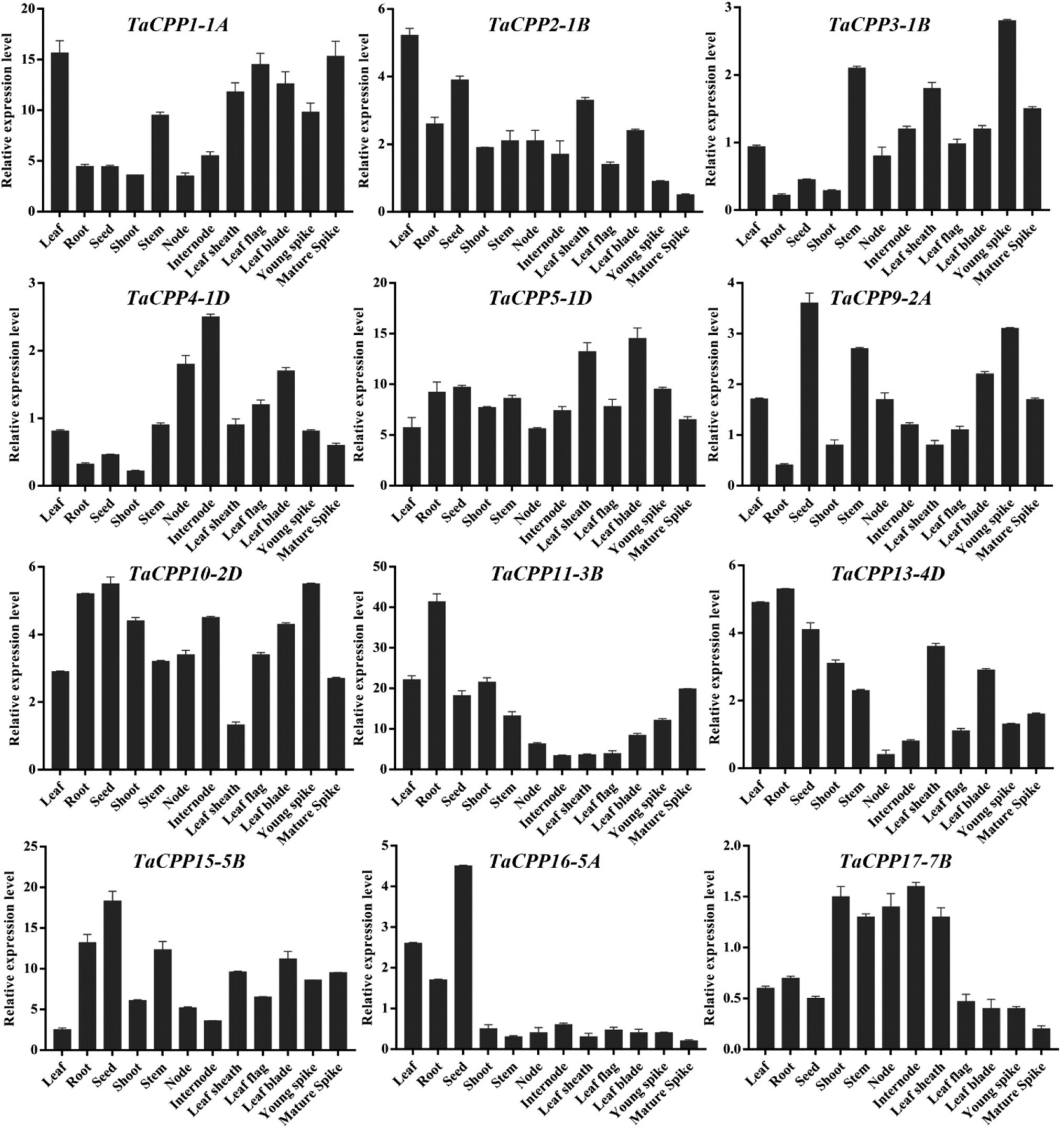
The expression analysis of *TaCPP* genes in different tissues, which were collected under normal conditions. Data of the quantitative RT-PCR analysis were presented in the form of the mean and standard deviation of three biological replicates of each biological sample. The *y*-axis is the relative expression level between two samples.

Thirty-nine different tissues were selected to explore TaCPP gene family expression using the GENEVESTIGATOR V3 database (https://genevestigator.com/gv/) [28]. We found that all the homologs of *TaCPP1*, *TaCPP12*, *TaCPP14*, and *TaCPP16* genes displayed high expression levels in the examined tissues (Figure S3). The *TaCPP2*, *TaCPP3*, *TaCPP4*, *TaCPP5*, *TaCPP6*, *TaCPP7*, and *TaCPP8* genes were less expressed in the tissues apart from meiocyte, microspore, stamen, and anther. Extremely low expression was noted for *TaCPP10-2D* and *TaCPP11-3A* in the tissues except microspore (Figure S3). Most of the wheat *CPP* gene family members were detected with high transcript levels in meiocyte, microspore, stamen, and anther. The rice *CPP* gene family members were highly expressed in almost all the tissues (Figure S3). The expression of the rice *CPP* gene family was higher than wheat *CPP* gene family members in all the tissues. The (qRT-PCR) found that each *CPP* gene family member displayed a high transcript level of at least in more than two tissues. We found that most of the *TaCPP* genes were diversely expressed in all the tested tissues (Figure 3). In leaf, *TaCPP* gene family members had highly expressed apart from *TaCPP3-1B*, *TaCPP4-1D*, *TaCPP9-2A*, and *TaCPP17-7B*. Most of the *TaCPP* gene family members display high expression levels in seed except *TaCPP3-1B* and *TaCPP17-7B*. Similarly, most of the *TaCPP* gene family members exhibited diverse expression levels in root and shoot. The *TaCPP1-1A*, *TaCPP3-1B*, *TaCPP5-1D*, *TaCPP9-2A*, *TaCPP10-2D*, *TaCPP11-3B*, and *TaCPP15-5B* were noted with high transcript levels, whereas the remaining *TaCPP* genes were low expressed in stem (Figure 3). The transcript level of *TaCPP2-1B*, *TaCPP3-1B*, *TaCPP9-2A*, *TaCPP13-4D*, *TaCPP16-5A*, and *TaCPP17-7B* displayed low expression levels in node, internode, and mature spike. Furthermore, some *TaCPP* genes, such as *TaCPP1-1B*, *TaCPP2-1B*, *TaCPP5-1D*, *TaCPP10-2D*, and *TaCPP15-5B*, have highly expressed in leaf-related tissues. Based on the expression fold, we found that *TaCPP11-3B* was the most expressed, followed by *TaCPP15-5B*, *TaCPP1-1B*, and *TaCPP5-1D*, whereas *TaCPP17-7B* exhibited low expression (Figure 3).

### 3.5 Expression analysis of *TaCPP* gene family under hormonal stresses

**Figure 4:**
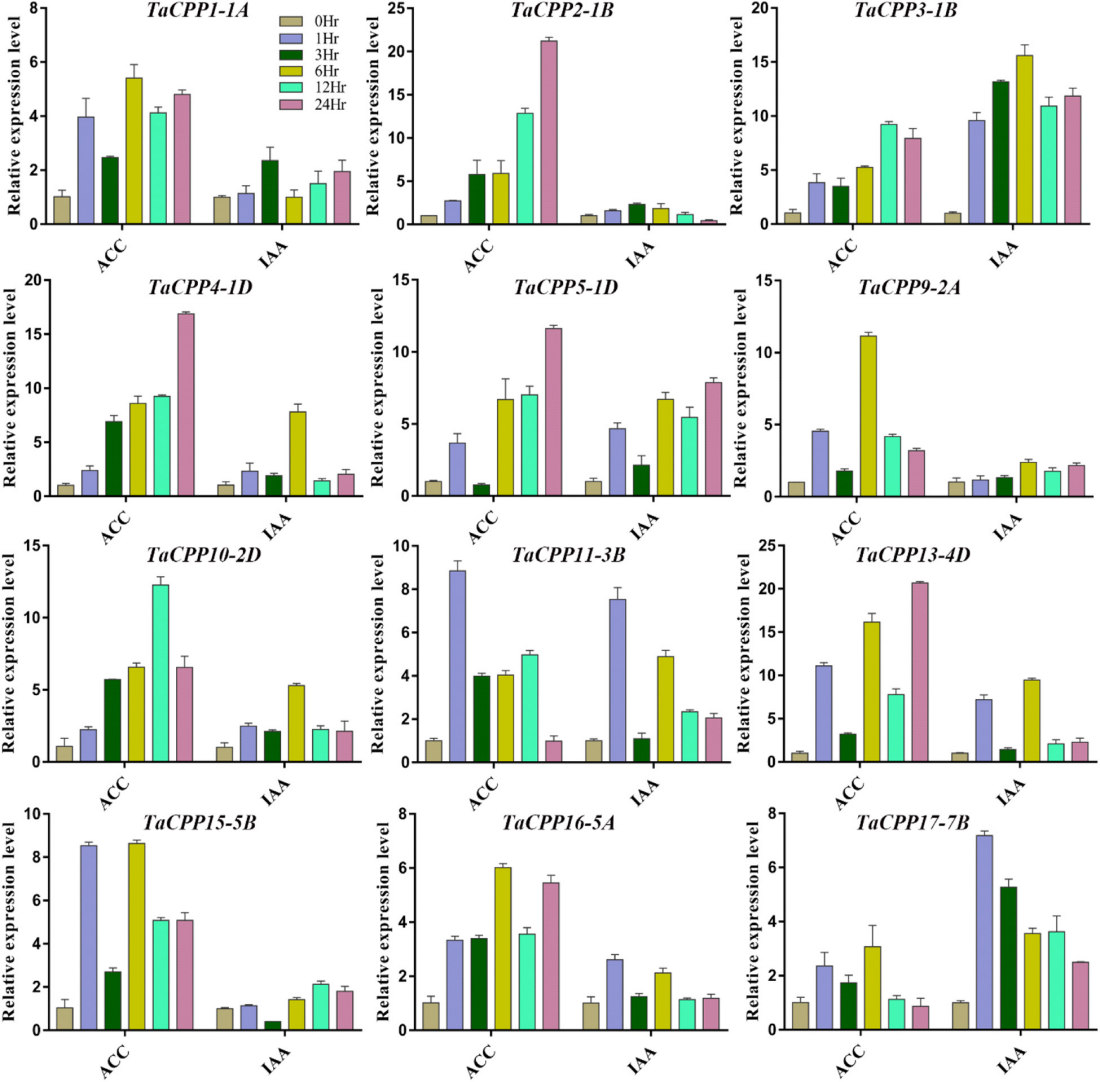
The expression analysis of *TaCPP* genes under ACC and IAA treatments with extending period from 0 to 24 h. Data of the quantitative RT-PCR analysis were presented in the form of the mean and standard deviation of three biological replicates of each biological sample. The *y*-axis is the relative expression level between two samples.

It has been reported that a few microbes and plants synthesize IAA, which plays a crucial role in shoot and root developments [32,33,34]. The ACC is recorded with function as a signal itself under stress conditions. Therefore, several mysterious questions are debatable about their function in extending the period. Here, we evaluated the transcript level of *TaCPP* gene family members under IAA and ACC stress to examine the role of *TaCPP* gene family members in growth and development (Figure 4). In response to exogenous ACC, we found that the transcript level of most of the *TaCPP* gene family members was promoted at different time points apart from the *TaCPP17-7B* gene. Most of the *TaCPP* gene family members were upregulated at the 24 h time point excluding the *TaCPP11-3B* gene. At 6 and 12 h time points, all the *TaCPP* gene family members showed high expression levels under ACC stress conditions (Figure 4). The transcript of the studied *TaCPP* gene family members was low at 1 and 3 h time points. Under exogenous IAA treatment, we found quite a mixed transcript level for the examined *TaCPP* gene family members (Figure 4). For instance, some *TaCPP* genes, such as *TaCPP1-1A*, *TaCPP2-1B*, *TaCPP9-2A*, *TaCPP15-5B*, and *TaCPP16-5A*, exhibited low transcript levels, while the remaining *TaCPP* gene family members showed high expression levels under IAA stress conditions. The transcript level of the *TaCPP3-1B* gene was high at all the time points and displayed high fold expression compared with other *TaCPP* gene family members in response to exogenous IAA treatment (Figure 4). The high expression for most of the *TaCPP* gene family members was noted at 24 h time points, followed by 1 and 6 h time points, respectively.

### 3.6 Expression analysis of *TaCPP* gene family under abiotic stresses

**Figure 5:**
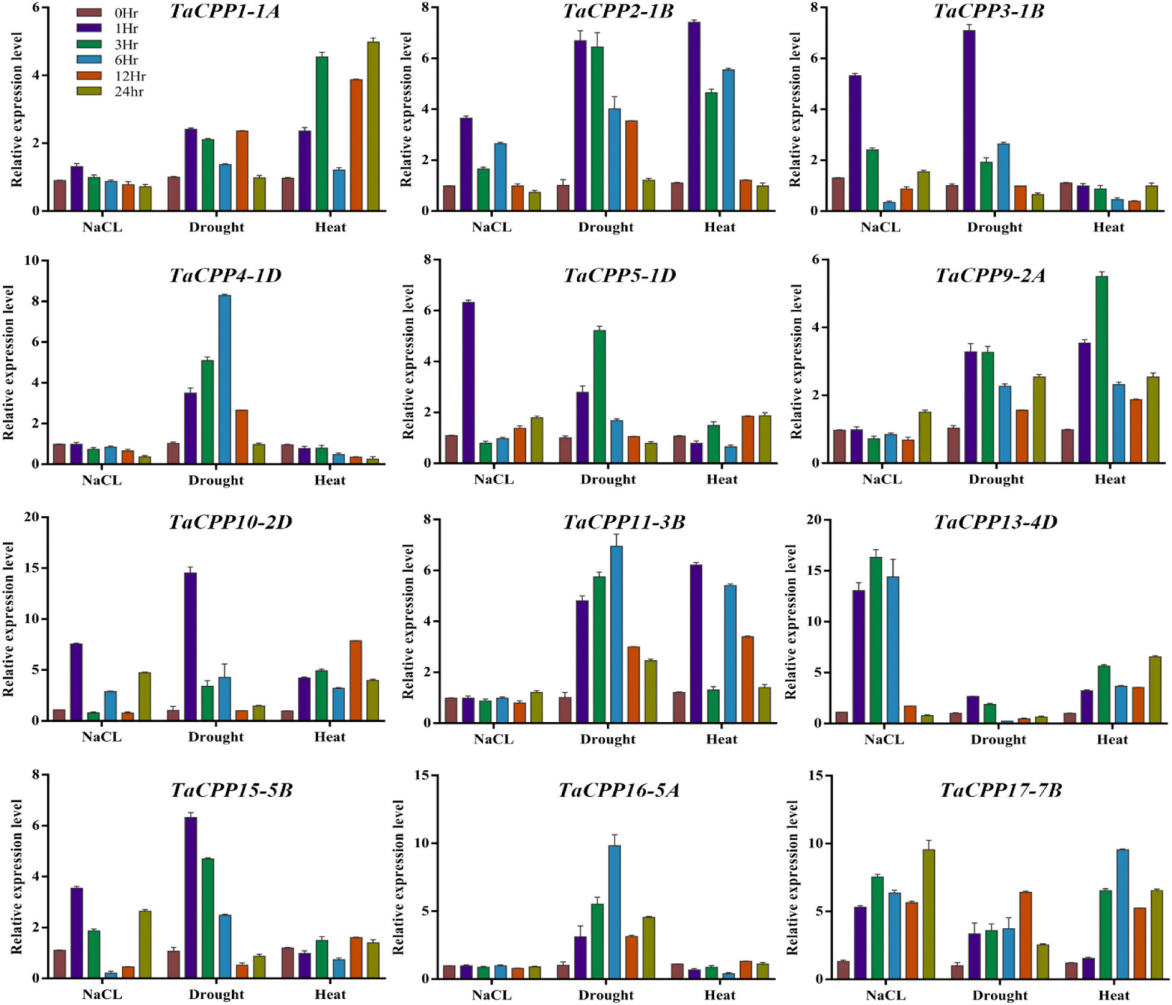
The expression analysis of *TaCPP* genes under heat, drought, and NaCl treatments with extending the time point from 0 to 24 h. Data of the quantitative RT-PCR analysis were presented in the form of the mean and standard deviation of three biological replicates of each biological sample. The *y*-axis is the relative expression level between two samples.

It has been reported that gene expression analysis can provide important information regarding gene function; therefore, we used qRT-PCR to investigate the transcript level of the wheat *CPP* genes under diverse conditions, such as drought, cold, and salt, at different time points (Figure 5). Under treatment, more than a two-fold difference in the transcript level was considered to be true differences for a gene. Under salt treatment, we noted that most of the *TaCPP* gene family members displayed a low level of transcript level apart from *TaCPP5-1D*, *TaCPP3-1B*, *TaCPP13-4D*, *TaCPP17-7B*, and *TaCPP10-2D* genes. We found that most of the *TaCPP* gene family members were low expressed at all the time points, excluding the 1 h time point (Figure 5). We detected that the transcript level of *TaCPP1-1A*, *TaCPP2-1B*, *TaCPP3-1B*, *TaCPP4-1D*, *TaCPP5-1D*, *TaCPP9-2A*, *TaCPP10-2D*, *TaCPP11-3B*, *TaCPP15-5B*, and *TaCPP16-5A* was low, whereas the rest of the two *CPP* members was high under salt conditions. Only one *CPP* gene (*TaCPP11-3B*) had a high expression profile under drought stress compared to the 0 h sample (control), whereas TaCPP 13-4D and TaCPP5-1D had a high expression profile at all time points a low expression profile. Under drought stress, the expression of *TaCPP2-1B*, *TaCPP4-1D*, *TaCPP11-3B*, *TaCPP*, and *TaCPP17-7B* was high at 3 and 6 h time points, respectively, while the expression of other *CPP* members was upregulated at two or three points (Figure 5). Similarly, under heat stress, the transcript level of *TaCPP1-1A*, *TaCPP9-2A*, *TaCPP10-2D*, *TaCPP13-4D*, and *TaCPP17-7B* was high at all time points. Furthermore, several *CPP* members (*TaCPP3-1B*, *TaCPP4-1D*, *TaCPP5-1D*, *TaCPP15-5B*, and *TaCPP16-5A*) were downregulated, whereas the other four *CPP* genes were up- and downregulated at different points. Consequently, we identified that most wheat *CPP* member transcripts were greatly influenced by heat and salt stresses. Furthermore, we observed that *CPP* member transcripts were up- and downregulated at different time points during drought conditions. All these results showed the role of the *CPP* gene family in plant growth and development and their response to multiple stresses.

### 3.7 Subcellular location and protein–protein interaction of CPP proteins

**Figure 6:**
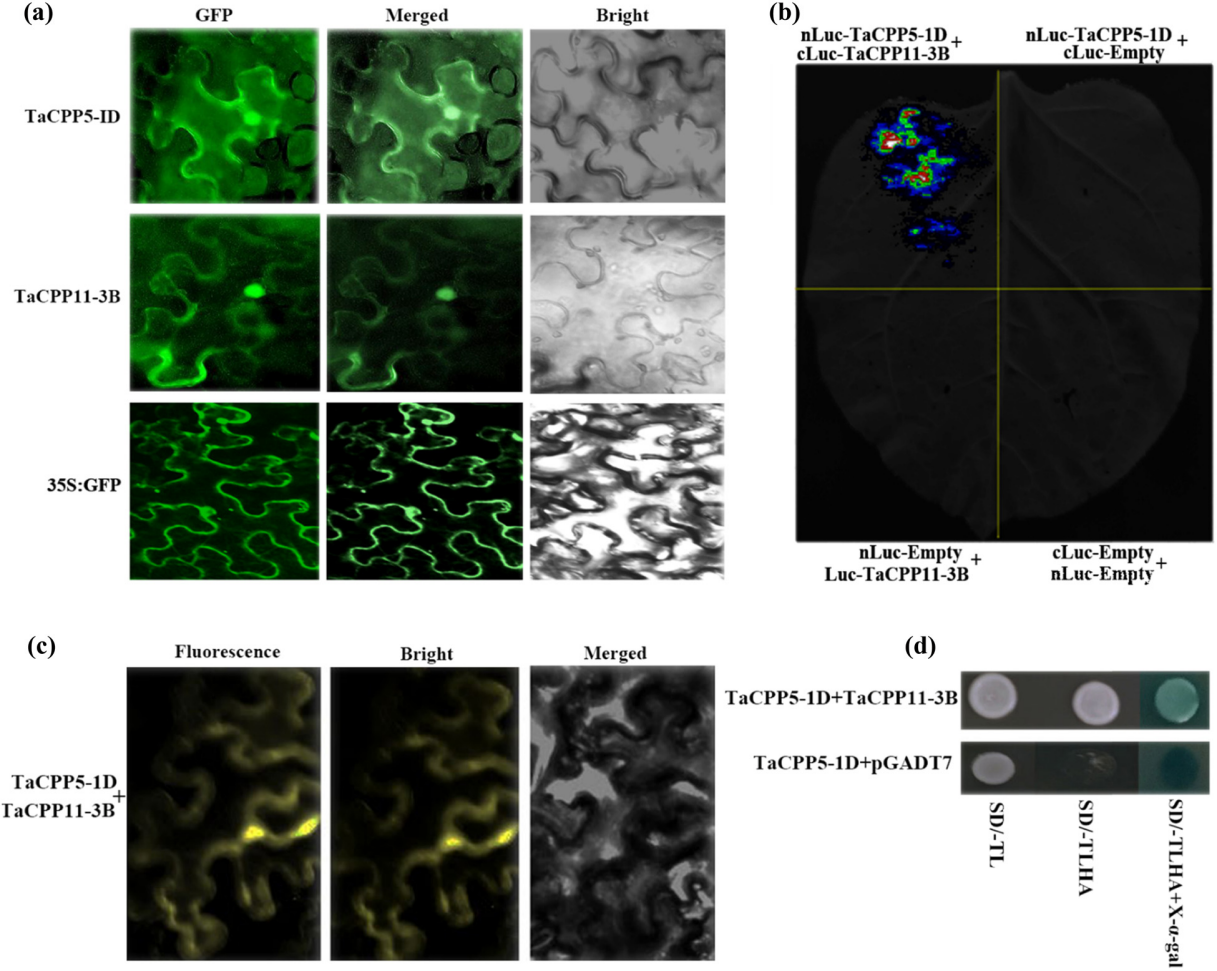
(a) The subcellular location of the CPP gene family members. The CDS region was ligated into the pCAMBIA-1302 vector and the constructed vector was then overexpressed in tobacco leaves. The GFP-fusion protein signals were detected using the confocal microscope. (b) The interaction of TaCPP5-1D and TaCPP11-3B proteins using firefly LCI assay. (c) The interaction of TaCPP5-1D and TaCPP11-3B proteins using the BiFC assay. (d) Yeast two-hybrid system confirmation of TaCPP5-1D and TaCPP11-3B proteins’ interaction.

The full-length CDS of *TaCPP5-1B* and *TaCPP11-3B* genes were cloned into the pCAMBIA-1302 vector. The vectors were then infiltrated into tobacco leaves using *A. tumefaciens*. After 48 h, we checked the *GFP* signal under the confocal microscope. Several studies have reported that most of the TFs are located in the nucleus of the cell. Here, we found that *TaCPP5-1B* and *TaCPP11-3B* proteins produced strong *GFP* signals in the nucleus (Figure 6a), suggesting that CPP TFs are also located in the nucleus of the cell and perform a diverse role in plant growth and development, particularly under hormonal and abiotic stresses. We used the protein–protein interaction assay to investigate the interaction of CPP proteins with other CPP proteins (Figure 6b). The firefly LCI assay found that TaCPP5-1D promoted the luciferase activity when TaCPP5-1D was co-expressed with TaCPP11-3B proteins in the tobacco leaves (Figure 6b). The interaction of TaCPP5-1D with TaCPP11-3B protein was confirmed by the bimolecular fluorescence complementation (BiFC) assay. We observed that the co-infiltration of TaCPP5-1D and TaCPP11-3B proteins produced strong fluorescence signals in tobacco leaves (Figure 6c). The LCI and BiFC results were further evaluated by the yeast two-hybrid system. The TaCPP5-1D and TaCPP11-3B proteins were co-expressed in yeast cells (Figure 6d). The yeast cells containing TaCPP5-1D and TaCPP11-3B proteins displayed significant growth on S/D media compared with control, indicating that TaCPP5-1D regulates the plant growth and development by interacting with TaCPP11-3B protein.

### 3.8 Overexpression of *TaCPP5-1D* genes positively regulates plant growth and development

**Figure 7:**
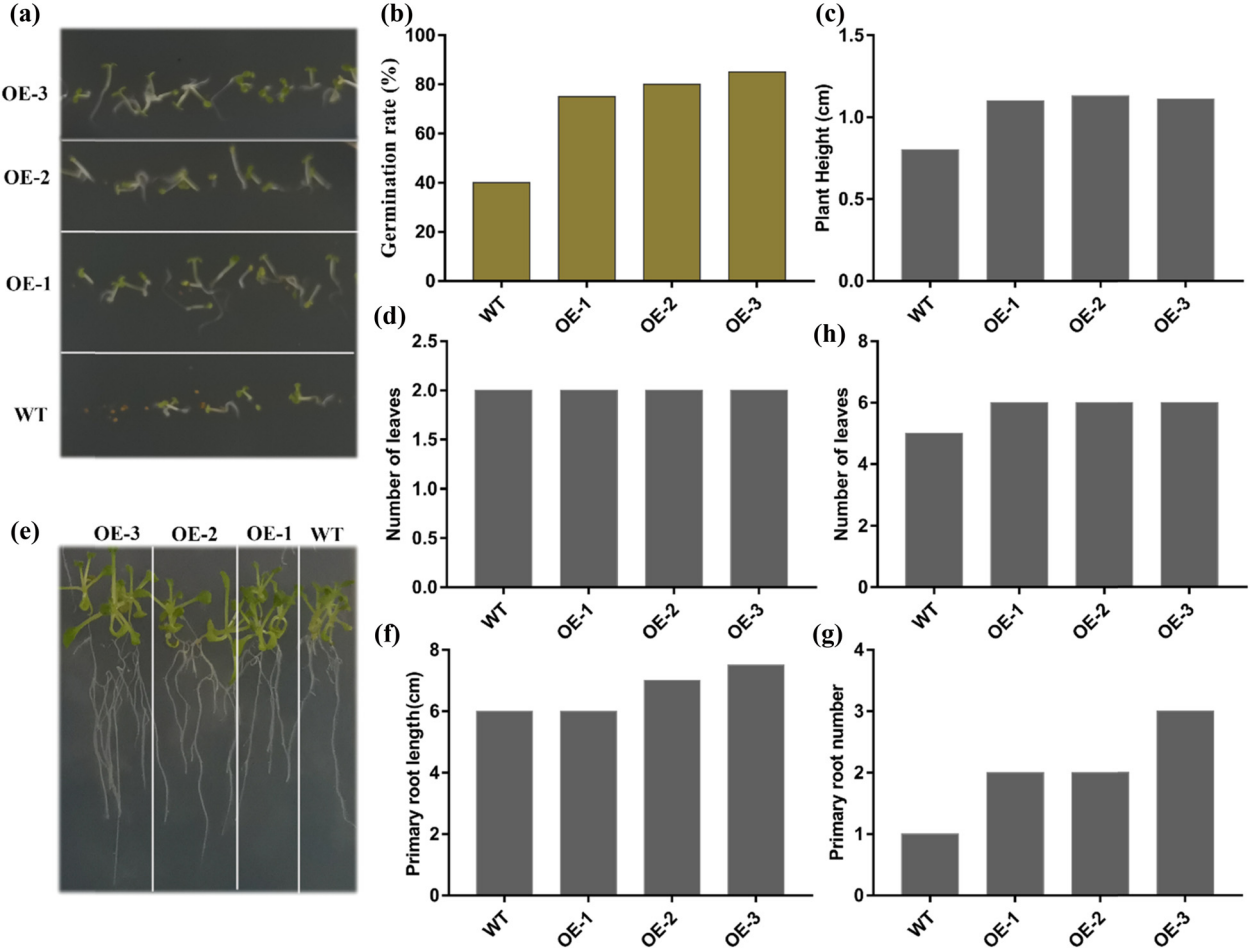
Phenotypic evaluation of Ta-CPP5-1D-overexpressed plants under 250 mM inositol. (a and b) Germination rate (%) of 1-week-old Ta-CPP5-1D-overexpressed and WT plants. (c) Shoot height of Ta-CPP5-1D-overexpressed and WT plants. (d) Average number of leaves per plant. (e) Root and shoot activities of 2-week-old Ta-CPP5-1D-overexpressed and WT plants. (f) Average primary root length. (g) Average primary root number. (h) Average number of leaves per plant.

To investigate the possible function of the *CPP* genes in plant growth and development, we overexpressed the *TaCPP5-1D* genes in *Arabidopsis*. We evaluate the function of the *TaCPP-1D* gene under osmotic stresses (250 mM inositol). In response to inositol, the *TaCPP5-1D*-overexpressed plants displayed a higher germination rate compared with WT plants (Figure 7a and b). The 1-week-old *TaCPP5-1D*-overexpressed plants showed a larger shoot size than WT plants (Figure 7c). However, no significant changes were observed in the number of leaves at this stage between 1-week-old *TaCPP5-1D*-overexpressed and WT plants (Figure 7d). Then, we evaluated the root and shoot activities of the 2-week-old *TaCPP5-1D*-overexpressed and WT plants under inositol stress conditions (Figure 7e). The *TaCPP5-1D*-overexpressed plants were noted with higher root activity than WT plants. The primary root length and number of *TaCPP5-1D* plant were higher than WT plants under 250 mM inositol condition (Figure 7f and g). Moreover, the number of leaves was more in *TaCPP5-1D*-overexpressed plants compared with WT plants at this stage (Figure 7h). Overall, we found that germination, root, and shoot activities were enhanced in *TaCPP5-1D*-overexpressed plants in response to an inositol stress condition, signifying that CPP proteins may positively regulate the plant growth and development under stress conditions.

### 3.9 Antioxidant activities and contents of antioxidant enzyme in *Ta-CPP5-1D*-overexpressed plants

**Figure 8:**
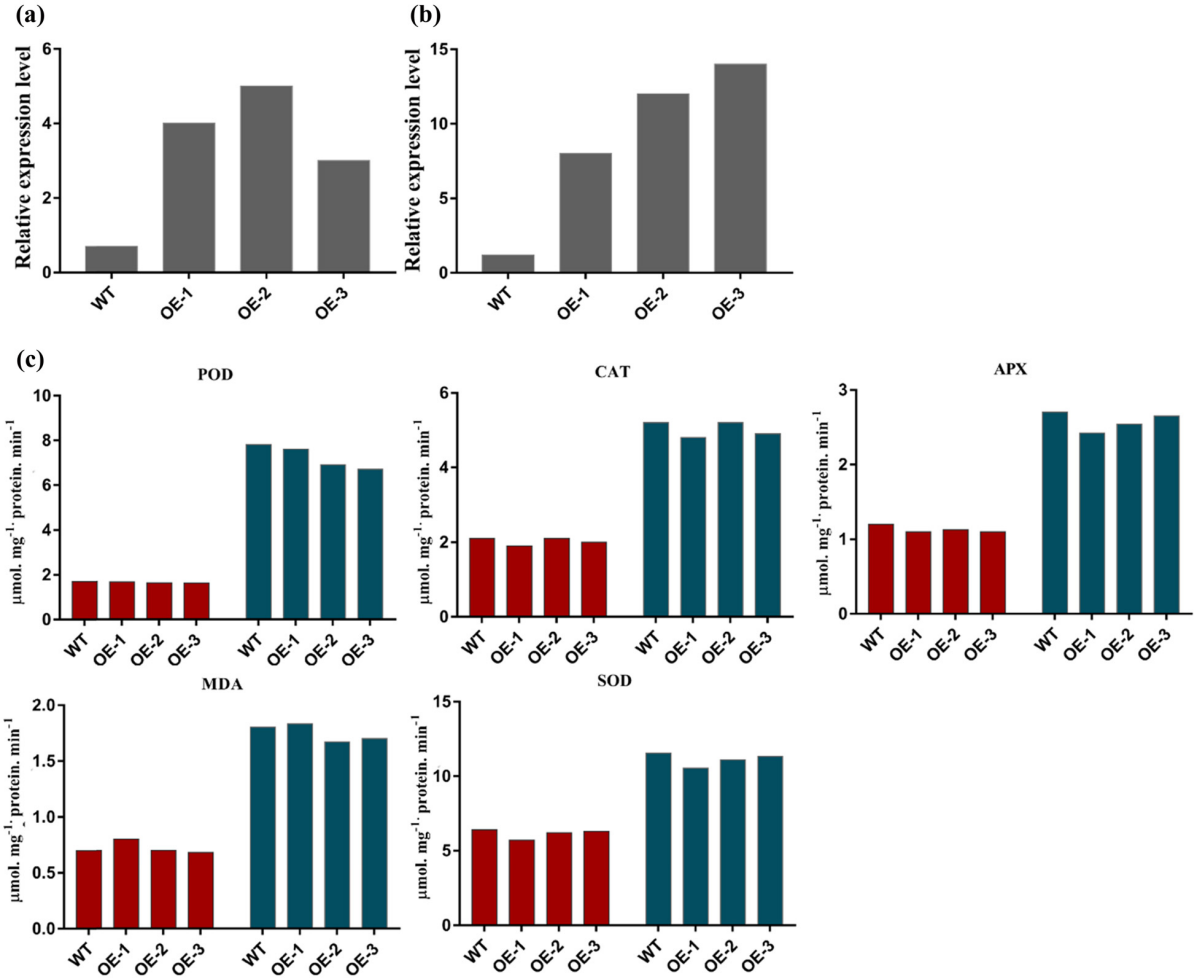
(a) Transcript level of TaCPP5-1D in the three independent homozygous T3 lines. (b) Transcript level of TaCPP5-1D gene in overexpressed plants under drought stress. (c) The antioxidant activities and contents of the antioxidant enzymes in *Arabidopsis*. The WT sample represents wild plants, and OE samples represent the TaCPP5-1D-overexpressed plants. Three-week-old *Arabidopsis* plants were exposed to drought stresses for the evaluation of the antioxidant analysis.

Furthermore, we evaluated the expression level of the *Ta-CPP5-1D* gene in the overexpressed and WT plants. The transcript level of the *TaCPP5-1D* gene was upregulated in overexpressed plants compared with the WT plants in response to drought stress (Figure 8b). We further investigated the antioxidant activities and contents of the various antioxidant enzymes under drought stress conditions. The POD and SOD activities were high in WT plants as compared with TaCPP5-1D overexpressed plants (Figure 8c). In *TaCPP5-1D*-overexpressed plants, CAT and APX showed lower activities compared with WT plants in response to drought stress. Similarly, the MDA content was also higher in WT plants than in *TaCPP5-1D*-overexpressed plants apart from the OE1 line (Figure 8c). Overall, we found that WT plants displayed high oxidant activities and contents, suggesting the less sensitivity of the *TaCPP5-1D*-overexpressed plants in response to drought stress. Based on these results, we predicted that *CPP* genes might play important role in plant growth and development under various adverse environmental stress conditions by regulating the antioxidant enzymes and various other physiological pathways.

## 4 Discussion

Plants are continuously exposed to different kinds of environmental stimuli throughout their life span. Therefore, plants respond to environmental stimuli by utilizing the activities of TFs, families of genes coding for specific TF proteins. Hence, it is of utmost importance to have comprehensive knowledge about the functions of these TF families that regulate multiple responses against abiotic stresses in main staple crops such as wheat (*T. aestivum* L.), rice (*O. sativa*), and maize [35]. CPP (cysteine-rich polycomb-like protein) proteins, whose typical character is the presence of CXC domains, are a member of a small TF family, widely present in plants and animals [4]. The *CPP* gene family has a crucial role in controlling cell division and the development of reproductive tissues [7,11]. Therefore, it is crucial to comprehensively assess the CPP protein’s role under unfavorable environmental stresses and key regulator hormones. In the present study, we performed a genome-wide identification, characterization, and expression analysis of the *CPP* gene family members under various hormonal and abiotic stresses.

### 4.1 CPP proteins in various plant genomes and their evolutionary relationship

In the model plants *Arabidopsis* and rice, the investigation of the *CPP* gene family members has been performed. Previous studies reported a total of 8, 11, 20, 17, 5, and 8 *CPP* gene family members in maize, *Arabidopsis*, rice, soybean, cucumber, and tea plants, respectively [1,2,17,18,36]. Previous studies had found lineage-specific expansion to have a crucial role in the differentiation and growth of the proteomes of multicellular eukaryotes [37,38]. And 80% of these results are demonstrated in the model plant *Arabidopsis thaliana* [38]. Here, we found that the small *CPP* gene family comprised 37 members in the wheat genome (Table 1), which were scattered and distributed in the genome (Figure S1b). Our results demonstrated that most of the *CPP* gene family members in plants might share a common ancestor during biological evolution. The phylogenetic analysis found that each subfamily possessed quite similar intron–exon, motif distribution, and physiological properties. For instance, the larger size and more introns–exons with motif distribution CPP proteins were found in subfamily-I (Figure 1 and Figure S1), whereas the medium size and normal intron–exon with the same motif distribution CPP proteins were detected in subfamily-III, and the smaller size CPP proteins with less number of introns–exons were noted in subfamily-II, -IV, and -V (Figure 1 and Figure S1), suggesting that *CPP* gene family member may share common features and ancestor during evolution. Recently, it has been reported that some gene families may originate from the common ancestral genes and split into different monocot and dicot species [39,40]. Previous studies reported that gene duplication is an important source of raw materials for genesis, and gene duplication can be divided into three principal evolutionary patterns, such as tandem duplication, segmental duplication, and transposition events likewise, replicative and retroposition [41]. Previous successful work had witnessed three rounds of whole-genome duplication in *Arabidopsis* [42,43]. Here, we found a total of four segmentally duplicated pairs of the *CPP* genes in *Arabidopsis* and three duplicated pairs in rice (Figure 2). Furthermore, we observed several segmentally duplicated pairs for *CPP* gene family members in the wheat genome (Figure 2), further signifying the importance of duplication in the expansion of gene families in plants. We further evaluated the gene structure of the *CPP* gene family members using the intron–exon distribution and motif arrangement in rice, wheat, and *Arabidopsis* (Figure S1). There was highly inconsistency in the number of exons and introns and the motif arrangement in the *CPP* gene family members in the studied plants (Figure S1b), indicating that the variation in the gene structure may play a vital role in performing the diverse role of the *CPP* gene family members in the growth and development.

### 4.2 CPP proteins could be important in plant stress biology

As we know that the gene expression profile can give clues about their functional features within developing plants. Therefore, we investigated the expression pattern of the *CPP* gene family members in rice and wheat. It has been reported that the *CPP* gene family members have been examined with a distinct feature in several plant species [3,5,17]. The microarray results demonstrated that higher expression of *CPP* gene family members was recoded within vegetative tissues; shoot apex, shoot, and root in wheat (Figure S3). These results further verify the previous finding on the *CPP* gene family role during cell division, growth, and development [5,17]. A previous study reported that the *CPP* gene family displayed distinct expression profiles in different parts of the plant [3]. For instance, it was found that *SOL2/TCX2* and *TSO1/AtCPP5* were less expressed in pollen and carpel than in other plants tissues [4]. The present study also found a similar expression pattern for most of the genes. For instance, the transcript of *TaCPP1-1A* and *TaCPP13-4D* genes was highly regulated in leaf but less expressed in mature spike (Figure 3). A quite similar expression level was also noted for *TaCPP16-5A* and *TaCPP13-4D,* which were highly expressed in seeds but a low transcript was recorded in leaf flag and young spike. It has been reported that IAA and ACC are considered the main regulators of plant growth and development through cell division, elongation, and tissue differentiation [44,45]. However, various studies also reported the independent role of ACC as a growth regulator under stress conditions [45]. In the present study, the qRT-PCR found that most of the *CPP* gene family members were highly expressed in response to ACC, particularly at 24 h time points (Figure 4), signifying the important role of the *CPP* gene family in plant growth and development in response to the high dose of ACC. In contrast, IAA significantly reduces the expression profile of *TaCPP1-1A*, *TaCPP2-1B*, *TaCPP4-1D*, *TaCPP9-2A*, *TaCPP10-2D*, *TaCPP11-3B*, *TaCPP13-4D*, *TaCPP15-5B*, *TaCPP16-5A*, and *TaCPP17-7B* by extending the period until 24 h (Figure 4). It has been reported that IAA could reduce the growth activities in plants [44]. Salt, heat, and drought stresses comprise osmotic and ionic homeostasis, growth regulation, and detoxification. TFs, such as the CPP-like family, have a crucial role in growth and development through cell division [3]. It was found that TFs had a specific influence on gene expression by altering the activity of a protein by either suppressing or promoting its function [46]. A recent study reported that the *CPP* gene family members displayed diverse expression levels in response to various abiotic stimuli in maize [18]. For instance, it has been noticed that the *ZmCPP7* gene displayed a high expression level under salt, heat, drought, and cold stresses suggesting the role of *CPP* genes in plant growth and development under stress conditions. In maize, most of the *CPP* gene family members were upregulated in response to heat stresses, further signifying the involvement of *CPP* genes in stresses [18]. Recently, it was noticed that the cucumber *CPP* genes were highly expressed in response to various abiotic and hormonal stresses [1]. It was observed that *CsCPP1*, *CsCPP3*, and *CsCPP4* were upregulated in response to cold stresses [1]. In tea plants, it was observed that the promoter of the *CPP* genes possessed the stress-responsive *cis*-elements, which regulate the expression of *CPP* genes under stress conditions [2]. Moreover, except for *GmCPP03* and *GmCPP07*, the remaining 18 *GmCPP* genes were all induced by heat shock under drought stress conditions, indicating that these genes are involved in the responses of soybean root systems to high-temperature stress and play important roles in regulating heat shock responses [17]. We found that most of the *CPP* gene family members were low expressed under salt stress apart from *TaCPP3-1B*, *TaCPP15-1D*, *TaCPP13-4D*, and *TaCPP17-7B*, whereas most of the *CPP* gene family members displayed high expression levels in response to drought and heat stresses (Figure 5), suggesting the important role of the *CPP* gene family in plant growth and development under unfavorable condition. Based on the previous and our current studies, we postulated that the *CPP* gene family may contribute to plant growth and development under various hormonal and abiotic stresses. Based on the previous and our current studies, we postulated that the *CPP* gene family may contribute to plant growth and development under various hormonal and abiotic stresses. Our results confirmed that CPP proteins bind with other CPP proteins to perform various functions in plant growth and development, particularly under adverse environmental stress conditions (Figure 6b–d). Furthermore, we found that the overexpression of *TaCPP5-1D* gene passivity regulates the germanium, shoot, and root activities in *Arabidopsis* (Figure 7). We further found that antioxidant activities and contents were lower in *TaCPP5-1D*-overexpressed plants compared with WT plants (Figure 8), signifying the less sensitivity of *CPP* genes and the importance of *CPP* genes in plant growth and development under various abiotic stresses.

## 5 Conclusion

In this study, we found a total of 37 and 11 *CPP* genes reported in wheat and rice, which were divided into five subfamilies based on the domain and structural distribution. Furthermore, the microarray and qRT-PCR analysis found the distinct expression pattern for most of the *CPP* gene family members in different tissues, suggesting that *CPP* gene family members may play a diverse role in plant growth and development. The qRT-PCR analysis of *TaCPP* genes under ACC, IAA, heat, drought, and NaCl treatments displayed varying transcript levels at different time points, indicating the vital role of the *CPP* gene family in plant growth and development under various abiotic and hormonal stresses. The overexpression of *TaCPP5-1D* detected that the CPP proteins might promote various plant developmental processes by targeting other CPP proteins under abiotic stress conditions. Thus, the present investigation provided important information for further functional studies on *CPP* genes in stress biology. Therefore, the current work could be used as primary knowledge to elucidate the regulation and pathway analysis of *TaCPP* TFs in plants.

## Supplementary Material

Supplementary Material
