# Supplementary material for: Genome-wide identification and expression analysis of CPP-like gene family in Triticum aestivum L. under different hormone and stress conditions
Source: Open Life Sci. 2022 May 18;17(1):544–62. doi: 10.1515/biol-2022-0051 (PMC9123298; doi:10.1515/biol-2022-0051)
Supplement: Supplementary Material [file biol-2022-0051-sm.pdf]

Supplementary materials

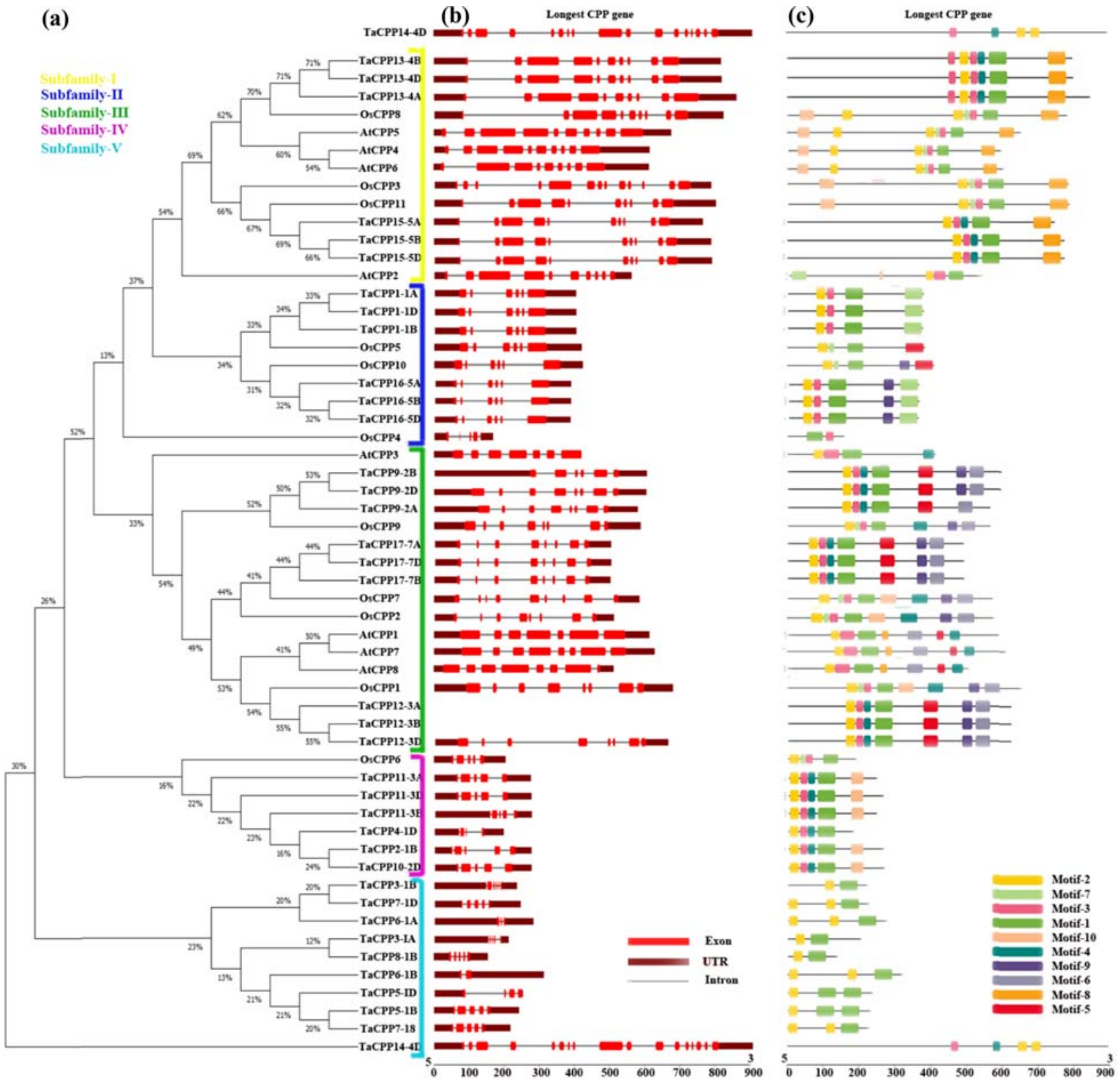

Figure S1: The evolutionary relationship of the CPP gene family members in rice, wheat, and Arabidopsis. (a) Phylogenetic relationships. (b) Gene structures. (c) Conserved motif analysis of *TaCPP, OsCPP, and AtCPP*-genes.

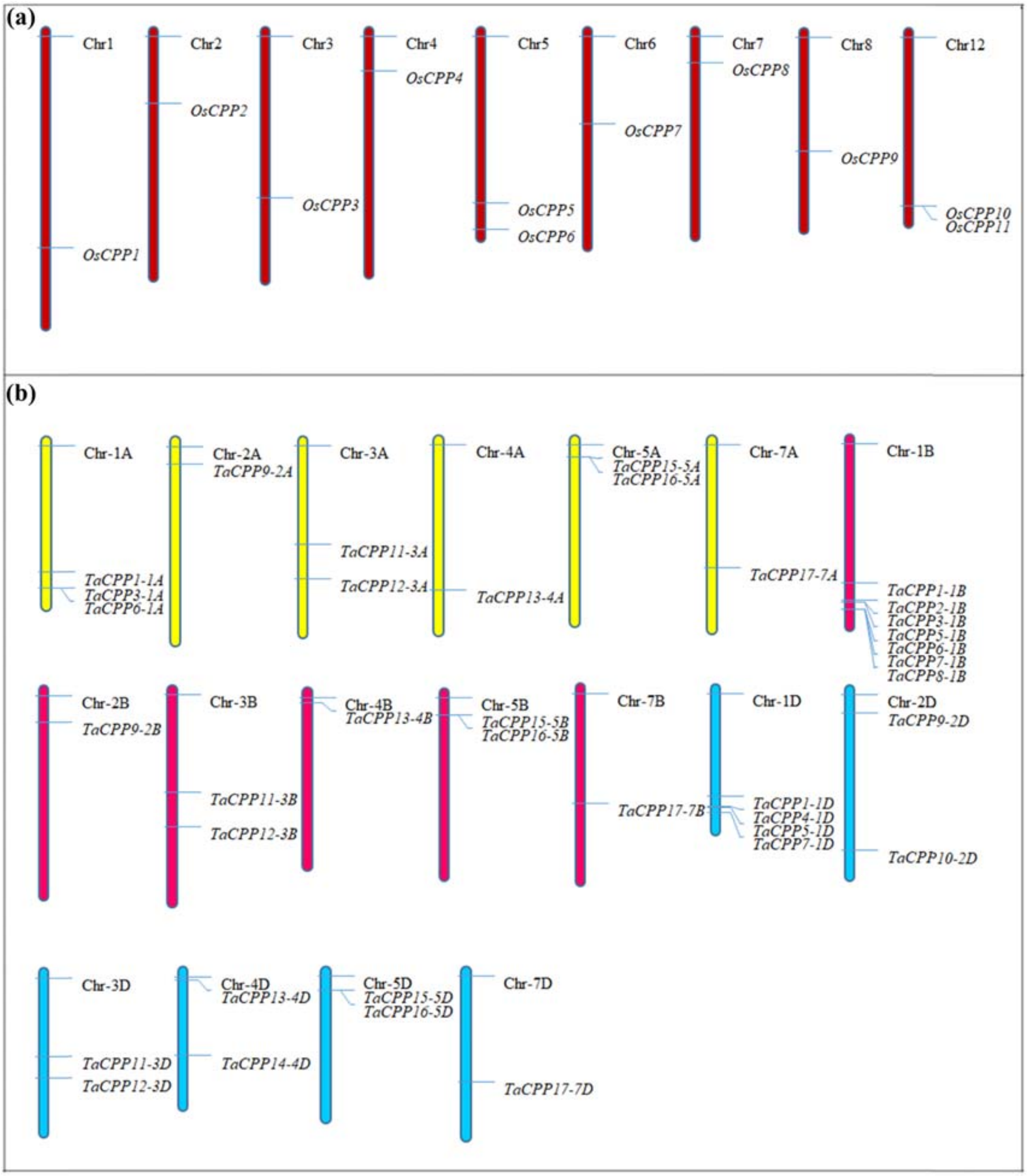

Figure S2: Chromosomal location of CPP gene family members in plants.

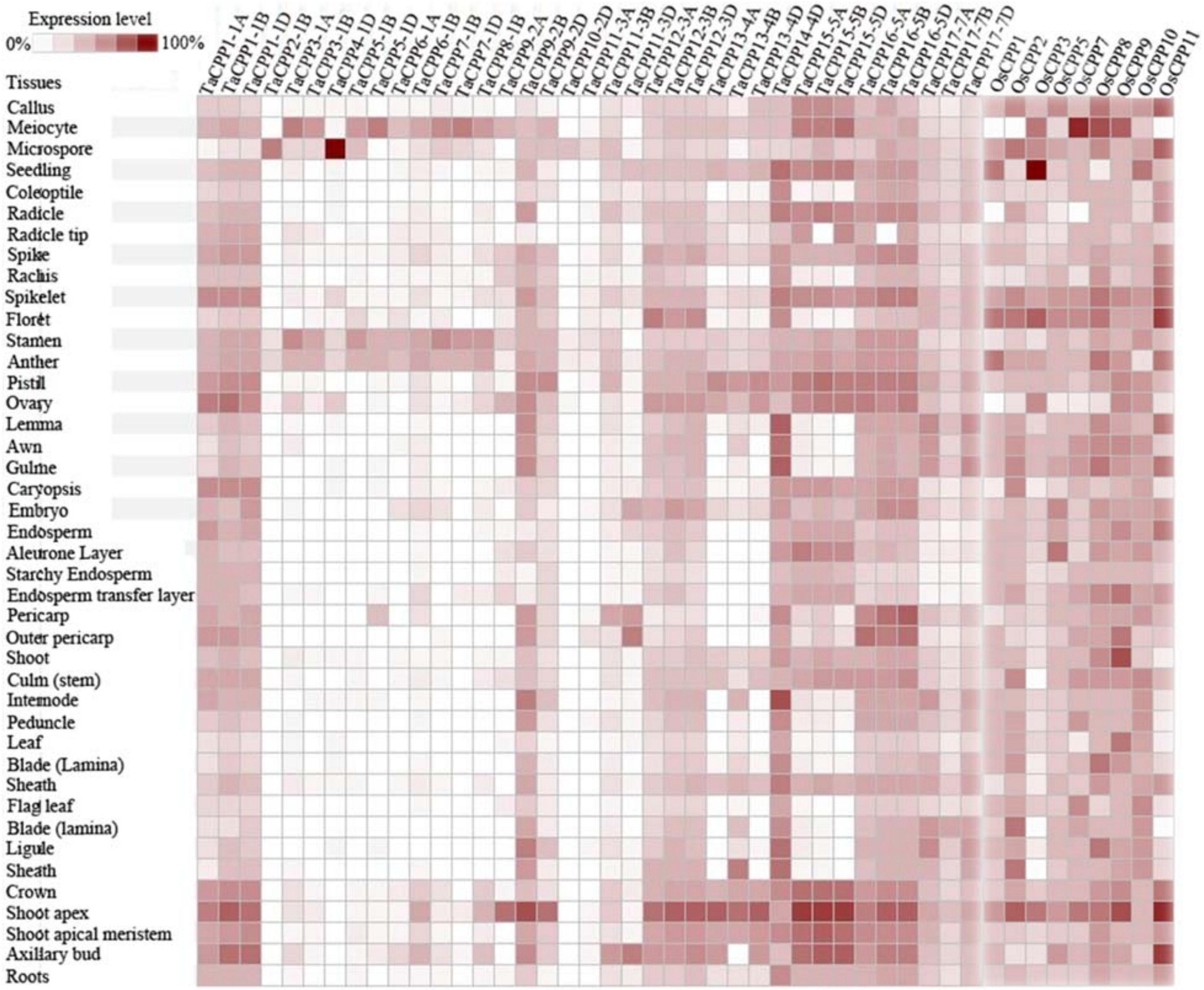

Figure S3: Tissues specific expression of CPP gene family members in plants.

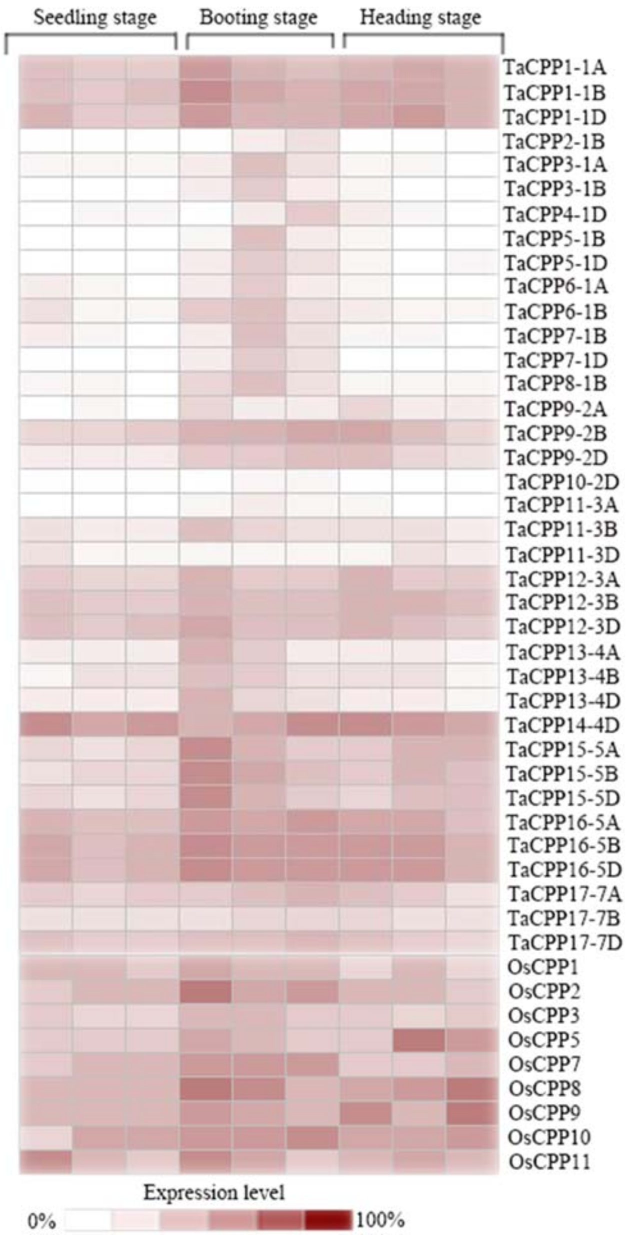

Figure S4: Expresssion profile of CPP gene family memebers at different developtmental stages in plants.

Table S1: Physiochemical properties of CPP gene family members in plants

Name	M.W	PI	Composition	GRAVY	AI	II
TaCPP1-1A	41.21	8.42	S(10.5), P(9.4), L(8.1)	-0.675	61.91	55.99
TaCPP1-1B	41.40	8.42	S(10.4), P(8.8), A(7.8)	-0.706	59.25	54.8
TaCPP1-1D	41.47	8.52	S(10.4), P(9.6), A(7.5)	-0.717	58.98	58.9
TaCPP2-1B	28.16	4.82	G(10.7), D(9.2), Q(8.4)	-0.572	61.49	40.16
TaCPP3-1A	21.69	9.32	G(12.4), S(10.4), C(8.9)	-0.297	60.84	40.45
TaCPP3-1B	22.42	7.62	C(13.2), G(11.4), A(8.7)	-0.391	47.35	32.03
TaCPP4-1D	19.37	5.05	C(10.6), G(10.0), A(7.2)	-0.623	47.22	45.82
TaCPP5-1B	24.30	8.91	C(11.8), A(11.0), G(8.8)	-0.724	39.96	52.05
TaCPP5-1D	25.15	8.61	C(12.0), A(11.6), K(9.4)	-0.727	44.12	58.58
TaCPP6-1A	31.12	8.07	A(12.2), S(10.9), C(9.2)	-0.262	59.7	49.85
TaCPP6-1B	31.97	8.49	A(25.0), C(8.5), Q(7.3)	-0.372	52.09	42.79
TaCPP7-1B	25.09	9.18	C(11.9), K(10.2), A(9.4)	-0.691	41.19	51.43
TaCPP7-1D	23.67	8.19	C(13.2), G(11.3), A(9.1)	-0.337	51.04	38.68
TaCPP8-1B	14.28	9.21	K(13.2), C(13.2), S(11.0)	-0.796	33.09	32.51
TaCPP9-2A	65.24	9.1	S(9.2), P(8.0), A(7.8)	-0.498	75.95	61.8
TaCPP9-2B	46.89	8.47	S(9.9), A(8.2), L(7.3)	-0.507	72.28	53.33
TaCPP9-2D	65.02	9.19	S(9.4), L(8.2),A(8.3)	-0.526	72.82	56.96
TaCPP10-2D	28.38	4.91	D(9.1), S(8.7), C(7.6)	-0.614	59.92	42.47
TaCPP11-3A	26.22	4.71	G(9.9), S(8.3), C(7.3)	-0.727	50.41	42.88
TaCPP11-3B	27.93	4.89	G(10.0), S(8.0), A(7.7)	-0.599	57.66	39.67
TaCPP11-3D	26.17	4.71	G(9.5), Q(7.8), A(7.0)	-0.673	52.22	40.77
TaCPP12-3A	66.73	9.1	A(9.4), P(8.6), K(7.5)	-0.585	68.57	49.13
TaCPP12-3B	66.77	9.14	A(9.3), P(8.3), K(7.5)	-0.571	67.77	48.47
TaCPP12-3D	66.71	9.1	A(9.6), P(8.8), G(7.3)	-0.583	68.26	49.5
TaCPP13-4A	90.60	7.75	S(12.1), L(7.5), K(7.3)	-0.568	64.78	55.63
TaCPP13-4B	84.92	6.65	S(12.3), G(7.4), A(6.6)	-0.689	59.48	57.81
TaCPP13-4D	85.02	8.55	S(12.5), K(7.5), L(7.0)	-0.65	61.6	57.49
TaCPP14-4D	98.82	7.91	S(11.0), R(7.3), L(7.2)	-0.729	63.16	54.4
TaCPP15-5A	82.04	7.6	S(12.2), L(6.9), K(6.8)	-0.614	65.31	55.94
TaCPP15-5B	82.11	7.78	S(12.0), L(6.9), K(6.8)	-0.613	65.31	56.81
TaCPP15-5D	79.45	7.61	S(12.6), L(6.9), K(6.8)	-0.659	63.06	58.24
TaCPP16-5A	39.64	8	S(9.2), P(7.8), R(7.3)	-0.71	58.46	50.48
TaCPP16-5B	40.02	8	S(8.9), P(8.6), R(7.2)	-0.716	57.79	52.71
TaCPP16-5D	39.89	8	S(8.9), P(8.6), R(7.2)	-0.709	58.13	53.9
TaCPP17-7A	52.83	7.46	A(9.7), P(8.8), S(7.4)	-0.738	59.12	53.36
TaCPP17-7B	52.71	8.12	A(9.5), P(8.2), S(7.6)	-0.714	60.62	54.93
TaCPP17-7D	52.95	7.75	A(9.3), P(8.4), S(7.8)	-0.74	60.1	54.28
OsCPP1	66.77	9.07	P(10.3), A(10.2), K(8.1)	-0.673	61.66	52.88
OsCPP2	55.19	8.57	P(8.9), K(8.2), S(8.0)	-0.789	61.29	67.7
OsCPP3	83.51	8.49	P(11..8), K(7.1), L(7.1)	-0.645	60.94	58.85
OsCPP4	16.43	8.77	S(13.6), A(8.8), R(7.5)	-0.428	58.37	65.03

(*Continued*)

Table S1: *Continued*

Name	M.W	PI	Composition	GRAVY	AI	II
OsCPP5	41.41	8.48	P(10.4), S(9.4), A(8.0)	-0.634	61.84	65.1
OsCPP6	21.63	6.69	S(12.4), C(9.1), N(8.2)	-0.877	39.28	54.89
OsCPP7	56.33	6.82	A(10.0), S(8.3), Q(7.1)	-0.771	56.45	55.1
OsCPP8	84.17	6.12	S(13.9), K(7.5), L(7.2)	-0.675	30.31	57.83
OsCPP9	65.70	8.78	S(10.4), L(9.0), A(8.7),	-0.446	74.75	49.35
OsCPP10	44.43	6.15	P(12.0), S(8.6), A(7.6)	-0.631	63.76	65.44
OsCPP11	82.02	6.61	S(13.0), P(8.0), L(7.5)	-0.638	63.78	60.14

Table S2: List of the primers that used in the present study

Name	Forwar primer	Reveres primer
TaCPP1-1A	GGTCGACGACTCGGGAGAGA	ACAGCTCCACCATGCCGTTC
TaCPP2-1B	GCACTGCACCTGCGAGAAGA	GAGGCCTCGAAGCAATGGCA
TaCPP3-1B	GGCTGGCCGACGAGGATTAC	CGTCACTTGTGCCACCCGTA
TaCPP4-1D	CTGCCGCAACACAGAGGACA	CCGGCGATGATCTTGGGCTT
TaCPP5-1D	TGCCTCGTGCAAGTGCCAAA	AGTCGCAGCGGTCCAAACAA
TaCPP9-2A	CCTGCTCAACCCGCAGCTAA	GGCGCAGTTGTACCCTTGGT
TaCPP10-2D	GCACTGCACCTGCGAGAAGA	GAGGCCTCGAAGCAATGGCA
TaCPP11-3B	CCTGGGTGTTCTGCGACGAG	CCGGCGATGATCTTGGGCTT
TaCPP13-4D	CGCTCAACGTCACCTCCGTT	GGTGCTAGTGCAGGCGAACA
TaCPP15-5B	CAGACAGCCTGAAAGGAGGG	CCGCTCTTGCAGATTGGAGA
TaCPP16-5A	CCCAGCGAGGACGGAACATC	CTTGTCCACCGGCGAAAGGT
TaCPP17-7B	GCCGGGATGACAGAAGCCAA	CCGGTGGTTGCTCCATGGTT
TaActin	GAAGTGCTTTTGAAGAGTCGGT	TTATTTCATACAGCAGGCAAGC
TaCPP5-1D-OE	GCTCTAGAATGTCGCAATTGCAGAAG	GGGGTACCTCCAACGCGTTTTGGCCG
TaCPP5-1D-nluc	GTCGACATGTCGCAATTGCAGAAG	GTCGACTCCAACGCGTTTTGGCCG
TaCPP11-3B-cluc	GGTACCATGGACGATTTGCCGCAC	GGATCCAATTCCACCGAGTTTGTT
